# The P2X_7_ Receptor Is Involved in Diabetic Neuropathic Pain Hypersensitivity Mediated by TRPV1 in the Rat Dorsal Root Ganglion

**DOI:** 10.3389/fnmol.2021.663649

**Published:** 2021-06-07

**Authors:** Anhui Wang, Xiangchao Shi, Ruoyang Yu, Bao Qiao, Runan Yang, Changshui Xu

**Affiliations:** ^1^Department of Physiology, Basic Medical College of Nanchang University, Nanchang, China; ^2^Medical Department, Queen Mary School, Nanchang University, Nanchang, China; ^3^Jiangxi Provincial Key Laboratory of Autonomic Nervous Function and Disease, Nanchang, China

**Keywords:** diabetic neuropathic pain, P2X_7_ receptors, TRPV1, dorsal root ganglion, satellite glial cells, neurons

## Abstract

The purinergic 2X_7_ (P2X_7_) receptor expressed in satellite glial cells (SGCs) is involved in the inflammatory response, and transient receptor potential vanilloid 1 (TRPV1) participates in the process of neurogenic inflammation, such as that in diabetic neuropathic pain (DNP) and peripheral neuralgia. The main purpose of this study was to explore the role of the P2X_7_ receptor in DNP hypersensitivity mediated by TRPV1 in the rat and its possible mechanism. A rat model of type 2 diabetes mellitus-related neuropathic pain (NPP) named the DNP rat model was established in this study. The mechanical withdrawal threshold (MWT) and thermal withdrawal latency (TWL) of DNP rats were increased after intrathecal injection of the P2X_7_ receptor antagonist A438079, and the mRNA and protein levels of TRPV1 in the dorsal root ganglion (DRG) were decreased in DNP rats treated with A438079 compared to untreated DNP rats; in addition, A438079 also decreased the phosphorylation of p38 and extracellular signal-regulated kinase 1/2 (ERK1/2) in the DNP group. Based on these results, the P2X_7_ receptor might be involved in DNP mediated by TRPV1.

## Introduction

Diabetic neuropathic pain (DNP) is one of the most common chronic complications of diabetes and one of the most common types of neuropathic pain (NPP) treated in the clinic (Galuppo et al., 2014; Lu et al., 2017; Pan et al., 2018). DNP, a type of chronic pain with a morbidity rate that is increasing annually, is associated with severe and long-lasting clinical symptoms that seriously affect patient quality of life. Additionally, effective clinical treatments are lacking, which has drawn increased attention to this difficult topic in the field of pain research in recent years (Vinik et al., 2013; Sloan et al., 2018).

The purinergic 2X_7_ (P2X_7_) receptor is an ion channel gated by the purine ATP involved in the transmission of pain information (Boue-Grabot and Pankratov, 2017; Burnstock, 2017; Ugur and Ugur, 2019). The release of ATP from the vesicles of injured neurons during acute injury further triggers the release of ATP from surrounding glial cells, thereby activating P2X_7_ receptors on adjacent glial cells and allowing glial cells to release ATP in an autocrine manner. Through this cascade reaction, the ATP signal is amplified (Volonté et al., 2012; Wei et al., 2016). The effect of a large dose of ATP on P2X_7_ receptors for an extended period of time promotes the formation and opening of plasma membrane pores and release of cytokines such as IL-1β, plasminogen and TNF-α, which further activate glial cells and aggravate neuronal damage (Zou et al., 2016; Janks et al., 2018). The P2X_7_ receptor is expressed on satellite glial cells (SGCs) of the dorsal root ganglion (DRG) (Kobayashi et al., 2013), and mechanical and thermal hyperalgesia are related to the activation of SGCs (Zou et al., 2019). Thus, the P2X_7_ receptor is involved in the pathogenesis of inflammation and NPP (Skaper et al., 2010; Ni et al., 2020).

Transient receptor potential vanilloid 1 (TRPV1), also known as the capsaicin receptor, is mainly distributed in medium- and small-diameter neurons of the DRG and the trigeminal ganglion (Kaneko and Szallasi, 2014) and plays an important role in enhancing of pain sensitivity caused by heat and chemicals in a model of diabetes-induced NPP (Gunthorpe and Szallasi, 2008; Hsu et al., 2014; Zhang et al., 2020). Moreover, primary sensory neurons play a key role in the process of pain generation and conduction, and TRPV1 is expressed in pain transmission pathways.

Studies have found that glial cells of the central nervous system could regulate the activity of spinal neurons and play a vital role in chronic pain (Milligan and Watkins, 2009; Tsuda et al., 2012; Ji et al., 2013). Studies have also shown that the activation of DRG SGCs is involved in NPP in some cases of peripheral neuropathy and that neurons in the sensory ganglia interact with glial cells (Gu et al., 2010; Jarvis, 2010). Therefore, the purpose of this study was to determine whether the P2X_7_ receptor plays a role in DNP mediated by TRPV1 in the rat DRG.

## Materials and Methods

### Animals and Ethics

Healthy and clean male Sprague-Dawley rats (weight, 180–220 g) were obtained from the Center of Laboratory Animal Science of Nanchang University, and the use of animals was approved by the Animal Protection and Use Committee of Nanchang University. All rats were housed in a clean room (22 ± 2°C, 50% relative humidity, and a 12-h light/12-h dark cycle) according to the Animal Care and Use Guidelines developed by the Ethics Committee of Nanchang University and provided free access to food and water and carried out in accordance with the National Institute of Health Guide for the Care and Use of Laboratory Animals.

### Animal Modeling and Groups

Rats were fed a high-sugar and high-fat diet (the high-sugar and high-fat diet was composed of 77.8% ordinary feed, 2% cholesterol, 0.2% sodium cholate, 10% lard, and 10% sucrose mixed with water, kneaded into balls, and placed in a constant temperature oven for baking) for 4 weeks and then intraperitoneally injected with a solution of streptozotocin (35 mg/kg), which can cause impaired pancreatic β-cell function. One week after the injection of streptozotocin, rats with a fasting blood glucose level > 7.8 mM or a non-fasting blood glucose level > 11.1 mM accompanied by a marked increase in behavioral sensitivity to pain were identified as successful DNP models.

In this experiment, the rats were divided into a control group (control group), DNP model group (DNP group), P2X_7_ receptor antagonist A438079 (S7705; Selleckchem)-treated DNP group (DNP + A438079 group), and normal saline (NS)-treated DNP group (DNP + NS group). Each group contained six rats and all rats participated in all procedures. The rats in the control group were not administered any treatment, the rats in the DNP + A438079 group were intrathecally injected with A438079, the rats in the DNP + NS group were injected intrathecally with an equal volume of NS, rats of the DNP + A438079 group and DNP + NS group were given A438079 (10 μM, 10 μl; dissolved in NS) and NS (10 μl) intrathecal injection once a day for 7 days (Ying et al., 2014), respectively. All treatments were administered starting when all experimental rats had become DNP model rats (namely, the first day of the eighth week). After the A438079 treatment, the blood glucose levels in type 2 diabetic rats were decreased compared with those in the untreated type 2 diabetic rats. Besides, a total of 20 rats were successfully modeled with diabetes. After successful modeling, two rats died during the observation period. Therefore, the success rate of DNP modeling is about 90%. After the last behavioral test, the rats were euthanized and the L4-6 DRGs of all experimental rats were isolated for later experiments.

Intrathecal injection was performed as described to previous studies (Mestre et al., 1994), and it is briefly described as follows: the rats were anesthetized with intraperitoneal injection of chloral hydrate with a volume fraction of 10% (30 mg kg^–1^); one hand was positioned at the spinous process of L4-6, and the other hand held a 25 μl micro sampler to pierce the subarachnoid space; there was a clear sense of breakthrough, and the needle was continuously injected until a typical rat tail lateral shake and jitter was considered as the puncture was successful.

### Mechanical Withdrawal Threshold (MWT)

Rats from each group were placed on a stainless-steel wire mesh (1 × 1 cm grid) in a transparent plastic chamber (22 × 12 × 22 cm) for 30 min. The environment was kept quiet, and the room temperature was maintained at 20–25°C. The rats were tested after adapting to the environment and exhibiting a quiet state (rats were tested at the same time of day). An electronic mechanical pain meter (BME-404; Tianjin, China) was used to test pain hypersensitivity thresholds. A filament with a random bending force was applied to the bottom of the left hind paw of rats from each group (the center of the foot was stimulated, the location of each stimulation was consistent throughout the experiment, and the intensity gradually increased) until each rat showed a significant contraction response of the stimulated foot. The test was repeated 6 times with an interval of 10 s between each test. The mechanical reflex threshold was read, and the average of 6 trials was recorded to obtain the final value (Liu C. et al., 2018).

### Thermal Withdrawal Latency (TWL)

The method for measuring the thermal withdrawal reflex latency of rats was similar to the procedure used to determine the mechanical withdrawal reflex threshold. Rats from each group were placed on a glass plate under a transparent bottomless square acrylic box (22 × 12 × 22 cm) for 30 min. The environment was kept quiet, and the room temperature was maintained at 20–25°C. The test did not start until the rats had adapted to the environment and exhibited a quiet state. An automatic thermal pain stimulator (BME-410C; Tianjin, China) was used to stimulate the midpoint of the left hind paw of the rat with a tungsten lamp. The light stimulus was approximately 0.5 cm in diameter to ensure a continuous thermal radiation stimulation for the specified duration. The length of time from the start of thermal stimulation to the point at which the rats raised their paws was observed, and the test was repeated 6 times with an interval of 2 min between each test. The thermal radiation stimulation time did not exceed 30 s. The time point at which each rat exhibit a contractile reflex was observed, the data were recorded, and the values from the 6 trials were averaged (Deng et al., 2018).

### Quantitative Real-Time Reverse Transcription Polymerase Chain Reaction

The DRG were isolated and then immediately placed in a glass homogenizer that had been soaked with prepared diethylpyrocarbonate. An appropriate volume of Transzol Up (ER501; TransGen Biotechnology Co., Ltd., Beijing, China) was added to every homogenizer and the DRGs were completely ground and lysed on ice. Then, total RNA was collected according to the instructions of the RNA extraction kit (ER501; TransGen Biotechnology Co., Ltd., Beijing, China). Next, total extracted RNA was reverse transcribed into cDNA using the First Strand cDNA Synthesis Kit (AE301; TransGen Biotechnology Co., Ltd., Beijing, China) according to the manufacturer’s instructions (a PCR amplification apparatus was used to incubate the samples at 42°C for 15 min and then heat them to 85°C for 5 s to deactivate the EasyScript^®^ RT/RI). At the same time, a NanoDrop2000 ultraviolet spectrophotometer (Thermo Fisher Scientific Inc., United States) was used to detect the cDNA concentration. The cDNA templates were mixed with Tip Green qPCR SuperMix kit reagent (AQ141; TransGen Biotechnology Co., Ltd., Beijing, China) in a 20-μl reaction, and qPCR was performed using an ABI PRISM system with the help of the StepOnePlus real-time PCR system (Applied Biosystems, Inc., Foster City, CA, United States). PCR specificity was determined by constructing a fusion curve, and the results were analyzed using the aforementioned software. Using the 2^–ΔΔ*CT*^ data analysis method, the average threshold cycle (CT) values of the target genes (TRPV1 and P2X_7_) were normalized to the average CT value of the internal control gene (β-actin) to calculate ΔCT values (ΔCT = CT target-CT internal reference), and the ΔCT value of the control group was used to normalize the ΔCT values of the experimental groups to calculate ΔΔCT values (ΔΔCT = ΔCT experimental group-ΔCT control group); expression data are presented in terms of relative mRNA quantity (RQ; RQ = 2^–ΔΔ*CT*^) (Liu et al., 2017). The sequences of the primers were as follows: TRPV1 forward, 5′-ATGACACCATCGCTCTGCTC-3′, and reverse, 5′-GTGCTGTCTGGCCCTTGTAG-3′; and β-actin forward, 5′-CCTAAGGCCAACCGTGAAAAGA-3′, and reverse, 5′-GGTACGACCAGAGGCATACA-3′.

### Western Blotting

The DRG were isolated and immediately rinsed in ice-cold PBS, and DRGs were placed in a pretreatment homogenizer to be mechanically dry-milled and crushed until homogenized. Next, an appropriate volume of protein lysis buffer [50 mM Tris-HCl (pH 7.4), 150 mM NaCl, 0.1% sodium dodecyl sulfate (SDS), 1% Nonidet P-40 (NP-40), 1% protein phosphatase inhibitor, and 1% protease inhibitor] was added to each sample, and the samples were then placed on ice for 30 min to ensure complete lysis. Afterward, the homogenates were centrifuged at 12,000 × g for 10 min at 4°C, the volume of supernatant was measured, and the supernatant was transferred to a new centrifuge tube. An aliquot of the supernatant was used to measure the protein concentration; then, an appropriate amount of protein loading buffer (DL101; TransGen Biotechnology Co., Ltd., Beijing, China) was added to the remaining supernatant, and the mixture was boiled in a 95°C water bath for 5 min to denature the proteins. The protein samples were separated on SDS-PAGE gels and then transferred to polyvinylidene fluoride membranes. The transfer time was adjusted according to the molecular weight of the protein. Afterward, each polyvinylidene fluoride membrane was incubated with a 5% blocking solution to block non-specific binding sites. The membrane subsequently was incubated with the following primary antibodies: rabbit anti-TRPV1 (Abcam, United Kingdom, 1:500); mouse anti-β-actin (TA-09; Beijing Zhongshan Biotech CO., CN, 1:800); rabbit anti-ERK1/2 (4695S; Cell Signaling Technology, United States, 1:1,000), rabbit anti-p-ERK1/2 (4370S; Cell Signaling Technology, United States, 1:1,000); rabbit anti-p38 (8690S; Cell Signaling Technology, United States, 1:800), rabbit anti-p-p38 (4511S; Cell Signaling Technology, United States, 1:800); rabbit anti-IL-1β and mouse anti-IL-10 (Abcam, United States, 1:500). Then, the membrane was incubated with a horseradish peroxidase-conjugated goat anti-rabbit or goat anti-mouse antibody. Finally, the membrane was subjected to exposure with hypersensitive ECL luminescent solution (FD8020; Fude Biotechnology Co., Ltd, Hangzhou, China) and signal detection. The integrated optical density (IOD) values of the detected proteins were analyzed by Image-Pro Plus software (Media Cybernetics Inc., Rockville, MD) (Dan et al., 2020).

### Immunofluorescence Staining

Double immunofluorescence staining was performed to show the coexpression of TRPV1 and neuronal nuclear protein (NeuN) and the coexpression of P2X_7_ receptor and glial fibrillary acidic protein (GFAP) in the DRGs of rats from each group. The L_4__–__6_ DRGs were fixed in 4% paraformaldehyde solution for 24 h. Then, the DRGs were removed and placed in 20% glucose solution overnight for dehydration at 4°C. The DRGs were embedded in OCT, and 8–10-μm-thick sections were prepared with a freezing microtome and subsequently fixed on slides at room temperature overnight before being stored at −20°C. Several slices were selected from each group and washed three times with 1 × PBS for 5 min each. The slices were fixed with 4% paraformaldehyde and then permeabilized with 0.3% Triton X-100 for 15 min (T8200; Beijing Solarbio Science Technology Co., Ltd.). Next, the slides were washed 3 times and dried. Afterward, a working solution of goat serum (ZLI-9022; Beijing Zhongshan Biotech CO., CN) was added dropwise to the ganglia to block non-specific binding sites and incubated in a water bath at 37°C for 1 h. Antibodies against TRPV1 (rabbit anti-TRPV1, 1:100, Abcam), NeuN (mouse anti-NeuN, 1:200), P2X_7_ (APR-004; rabbit anti-P2X_7_, 1:100, Israel Alomone) and GFAP (mouse anti-GFAP, 1:200) were diluted to the appropriate concentrations and then uniformly added to the slices dropwise. The slices were incubated at 4°C over night. The slices were brought to room temperature, washed 3 times and dried. Thereafter, secondary antibodies (TRITC-conjugated goat anti-rabbit, 1:200, Beijing Zhongshan Biotech Co., and FITC-conjugated goat anti-mouse, 1:200, Beijing Zhongshan Biotech Co., ZF-0312) were incubated with the sections at 37°C for 1 h. An antifluorescence quenching agent (AR1109; Boster Biological Technology Co., Ltd.) was applied, and then a coverglass was carefully placed on the slides. Finally, an inverted fluorescence microscope (Olympus, Tokyo, Japan) was used for imaging (Ge et al., 2019).

### Molecular Docking

AutoDock 4.2 was used for molecular docking experiments (Yang et al., 2019). Molecular docking is a computer simulation tool that attempts to predict the binding mode and affinity of ligands at the active sites of proteins and is a theoretical method used to simulate intermolecular interactions by selecting ligands that are close to the natural conformation and have the best affinity for the receptor through a scoring function. Molecular docking is also an important approach for structure-based drug design because it allows researchers to study the interactions between ligand molecules and receptor biomolecules and predict their affinity. The aim of docking technology is to determine the location of ligands in the binding sites in different directions and conformations to calculate the optimal binding geometry and energy. In this experiment, we used docking technology to determine whether two molecules have binding sites and bind one another, namely, whether A438079 bind to the TRPV1 receptor.

According the rat TRPV1 (PDB ID: 3J5P.1) protein sequence NP_114188.1 as target proteins, the structure of the TRPV1 protein 3D file was obtained by homology modeling via online SWISS-MODEL website^1^ (Hollopeter et al., 2001). A438079 (PubChem CID: 11673921) was used as the ligand. The protein and ligand structures were prepared using AutoDock Tools and Python scripts, named prepare_ligand4.py and prepare_receptor4.py, which are associated with the AutoDock 4.2 program. The binding pocket position in the target protein was specified with the AutoDock Tools molecular viewer. The parameters were maintained at their default values. Finally, the output files were viewed using MGL tools and PyMol^2^.

### Statistical Analyses

Statistical analyses were performed using Excel, SPSS 21.0 (IBM, United States) and GraphPad Prism 6.01 (GraphPad Software Inc., United States) software. The experimental data from each group are presented as the mean ± standard error of the mean (SEM). The data were analyzed by one-way analysis of variance (ANOVA) followed by Fisher’s least significant difference (LSD) test. For the behavioral test, two-way repeated-measures ANOVA followed by Bonferroni’s *post-hoc* test was carried out. The results were statistically significant when *P* < 0.05.

## Results

### Intrathecal Injection of A438079 Attenuated the Mechanical and Thermal Sensitivity of DNP Rats

**FIGURE 1:**
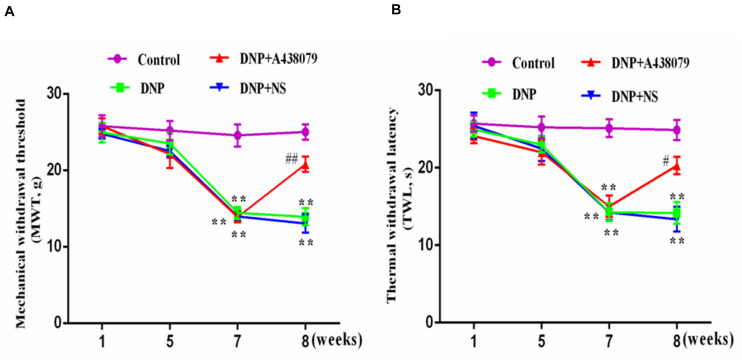
The effect of A438079 on the mechanical withdrawal threshold (MWT) and the thermal withdrawal latency (TWL) of DNP rats. **(A)** Changes in the MWTs of rats from each group. **(B)** Changes in the TWLs of rats from each group. The MWTs and TWLs of rats from the DNP group were significantly lower than the values of rats from the control group (namely, the sensitivity was increased). At the end of the 8th week, the MWTs and TWLs of rats in the DNP group treated with an intrathecal injection of the P2X_7_ receptor antagonist A438079 (DNP + A438079 group) were significantly higher than those of rats in the DNP group (i.e., decreased sensitivity). The data are presented as the mean ± SEM for the six animals in each group; compared with the control group, **P < 0.01; compared with the DNP group, ^#^*P* < 0.05, ^##^*P* < 0.01.

The MWT and TWL of the DNP group were lower than those of the control group (*p* < 0.01; Figures 1A,B); the MWT and TWL of DNP rats treated with the P2X_7_ receptor antagonist A438079 were higher than those of untreated DNP rats (*p* < 0.01), but a significant difference was not observed between the results obtained from the DNP group and DNP + NS group (*p* > 0.05). Thus, the P2X_7_ receptor antagonist A438079 reduced mechanical and thermal hyperalgesia in DNP rats, indicating that treatment with the P2X_7_ receptor antagonist A438079 may alleviate DNP in rats.

### A438079 Reduced TRPV1 mRNA Level in the DRGs of DNP Rats

**FIGURE 2:**
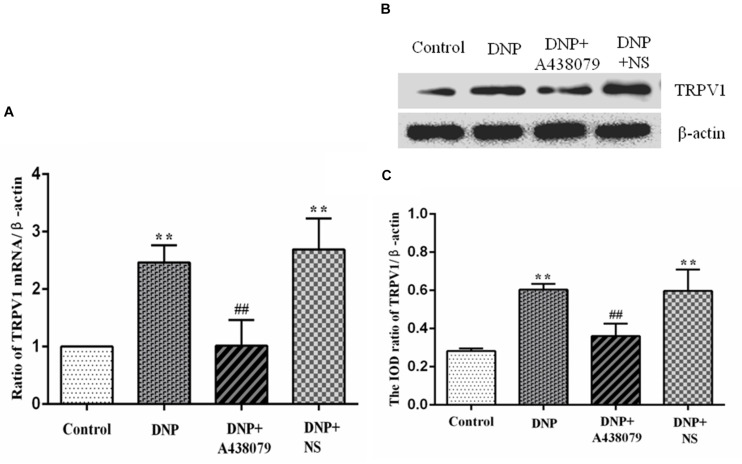
The levels of the TRPV1 mRNA and protein in the DRG were detected. **(A)** The level of the TRPV1 mRNA in the DRG from each group. **(B)** SDS-PAGE band of the TRPV1 protein in the DRG from each group of experimental rats. **(C)** Analysis of the relative level of the TRPV1 protein in the DRG from each group of experimental rats. Higher levels of the TRPV1 mRNA and protein were detected in the DRG in the DNP group than in the control group, while lower levels of the TRPV1 mRNA and protein were observed in the DRG in the DNP + A438079 group than in the DNP group. The data are presented as the mean ± SEM, *n* = 6; compared with the control group, **P < 0.01; compared with the DNP group, **^##^***P* < 0.01.

The mRNA level of TRPV1 in the DRGs of experimental rats in each group was detected by qPCR. A significantly lower level of the TRPV1 mRNA was detected in the control group than in the DNP group (*P* < 0.01; Figure 2A), but no obvious difference was detected between the control group and DNP + A438079 group (*P* > 0.05). A significant difference in the mRNA expression of TRPV1 was not observed between the DNP + NS group and DNP group (*P* > 0.05). Based on these results, A438079, a P2X_7_ receptor antagonist, might reduce the upregulation of TRPV1 mRNA in the DRGs of DNP rats.

### A438079 Decreased Level of the TRPV1 Protein in the DRGs of DNP Rats

Western blotting was performed to analyze protein levels in each group of experimental rats. Noticeably lower levels of the TRPV1 protein were observed in the control group than in the DNP group (*P* < 0.01; Figures 2B,C). In contrast, no fundamental difference in TRPV1 protein expression was observed between the control group and the DNP + A438079 group (*P* > 0.05). Expression of the TRPV1 protein was also not significantly different between the DNP + NS group and DNP group (*P* > 0.05). The above results demonstrate that A438079, a P2X_7_ receptor antagonist, might inhibit the increase in the levels of the TRPV1 protein in the DRGs of DNP rats.

### Coexpression of TRPV1 With NeuN and P2X_7_ Receptor With GFAP

**FIGURE 3:**
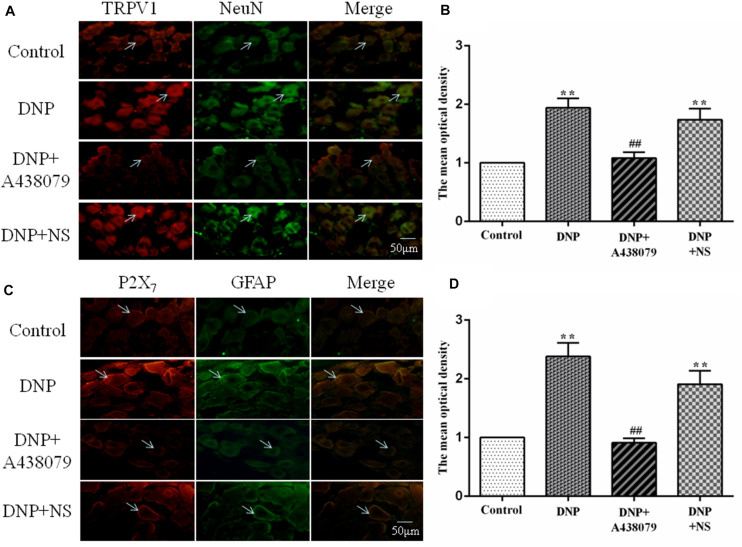
The coexpression of TRPV1 with NeuN and P2X_7_ receptor with GFAP was detected by immunofluorescence staining. **(A)** Levels of TRPV1 with NeuN coexpression in neurons in the rat DRG (*n* = 6 rats for each group). **(B)** The histogram shows the mean optical density of the coexpression of TRPV1 with NeuN. **(C)** Levels of P2X_7_ with GFAP coexpression in SGCs in the rat DRG (*n* = 6 rats for each group). **(D)** The histogram shows the mean optical density of the coexpression of the P2X_7_ receptor with GFAP. In the DNP group, the coexpression of TRPV1 with NeuN in the DRG was increased compared with that in the control group, while the coexpression of TRPV1 with NeuN in the DNP + A438079 group was decreased compared with that in the DNP group. Furthermore, the coexpression of P2X_7_ receptor with GFAP in the DRG was consistent with the coexpression trend described above. The green signal is fluorescein isothiocyanate (FITC)-labeled NeuN and FITC-labeled GFAP; the red signal is tetramethylrhodamine isothiocyanate (TRITC)-labeled TRPV1 and TRITC-labeled P2X_7_; and the yellow signal is the combination of the green and red signals. The arrow indicates activated neuronal cells and SGCs in the DRG. The scale bar represents 50 μm. The data are presented as the mean ± SEM; compared with the control group, **P < 0.01; compared with the DNP group, **^##^***P* < 0.01.

NeuN is a neuronal marker, a higher level of TRPV1 with NeuN coexpression was observed in the DNP group than in the control group. A lower level of coexpression was observed in the DNP + A438079 group than in the DNP group. A significant change was not detected between the DNP group and the DNP + NS group. Therefore, we speculate that the P2X_7_ receptor antagonist A438079 may inhibit coexpression of the TRPV1 receptor and NeuN in neurons in the DRG (Figures 3A,B).

Similarly, the marker for SGCs is GFAP, and its upregulation indicates the activation of SGCs. The coexpression of P2X_7_ with GFAP in the SGCs of each group followed a trend consistent with that for the coexpression of TRPV1 with NeuN in neuronal cells. Consequently, we concluded that the P2X_7_ receptor antagonist A438079 might inhibit the upregulation of the P2X_7_ receptor expression associated with the activation of SGCs in the DRG (Figures 3C,D).

### Effect of A438079 on the Levels of IL-1β and IL-10 in the DRGs of DNP Rats

**FIGURE 4:**
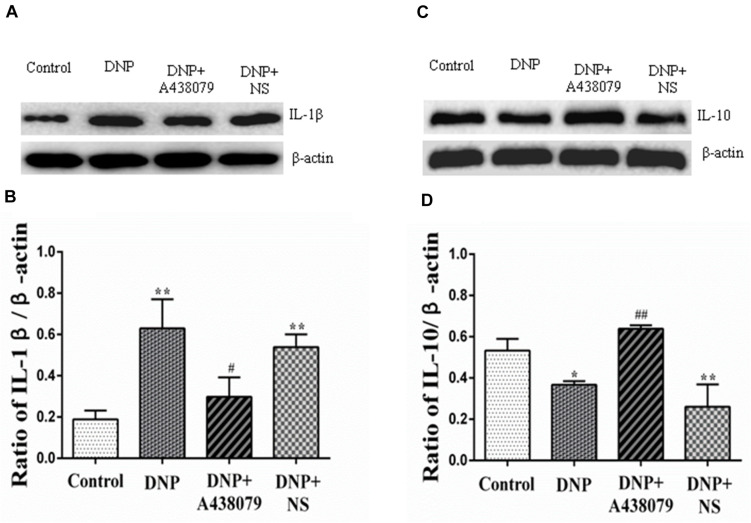
The relative levels of IL-1β and IL-10 in the DRG were detected by Western blotting. **(A)** SDS-PAGE band of IL-1β in the DRG in each group of experimental rats. **(B)** The relative levels of IL-1β in the DRG from each group of experimental rats. **(C)** SDS-PAGE band of IL-10 in the DRG in each group of experimental rats. **(D)** The relative levels of IL-10 protein in the DRG from each group of experimental rats. The relative level of the IL-1β protein in the DRG from the DNP group was higher than that in the control group, while a lower level of IL-1β was observed in the DRG from the DNP + A438079 group than in the DNP group. In contrast, the relative level of the IL-10 protein in the DRG from the DNP group was lower than that in the control group, while a higher level of IL-10 was detected in the DRG in the DNP + A438079 group than in the DNP group. The data are presented as the mean ± SEM, *n* = 6; compared with the control group, *P < 0.05, **P < 0.01; compared with the DNP group, **^#^***P* < 0.05, **^##^***P* < 0.01.

An increase in cytokine expression is another characteristic of SGC activation, and IL-1β released from SGCs is believed to initiate and maintain DNP. The IL-1β protein was expressed at significantly lower levels in the control group than in the DNP group (*P* < 0.01; Figures 4A,B) but a significant difference was not observed between the expression level in the DNP + A438079 group and control group (*P* > 0.05). A significant difference in the level of the IL-1β protein was not observed between the DNP + NS group and DNP group (*P* > 0.05). Based on the results described above, the P2X_7_ receptor antagonist A438079 may inhibit expression of the IL-1β protein in DNP rats.

IL-10, an anti-inflammatory cytokine, may provide protection for the body. The expression of the IL-10 protein was different from that of IL-1β protein. That is, the expression of IL-10 protein was decreased in the DNP group compared to the control group, and A438079 inhibited this downregulation (Figures 4C,D), suggesting that the P2X_7_ receptor antagonist A438079 may induce expression of the IL-10 protein in rats presenting a state of NPP.

### A438079 Inhibited the Phosphorylation of p38 and ERK1/2 in the DRGs of DNP Rats

**FIGURE 5:**
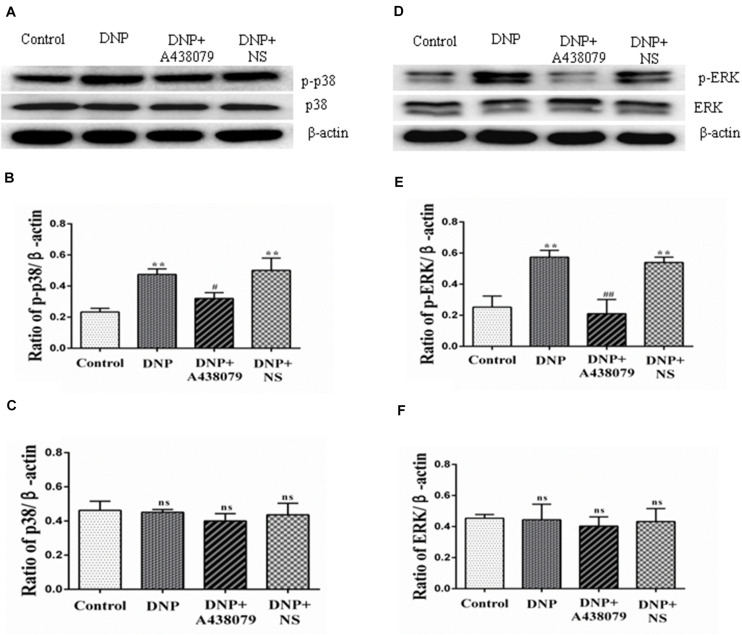
Western blotting was performed to detect the levels of proteins related to the p38 and ERK1/2 signaling pathways in the DRG. **(A)** SDS-PAGE bands of the p-p38 and p38 proteins in each group. **(B)** The ratio of the optical density of p-p38 to that of the internal reference. **(C)** The ratio of the optical density of p38 to that of the internal reference. **(D)** SDS-PAGE bands of p-ERK1/2 and ERK1/2 in each group. **(E)** The ratio of the optical density of p-ERK1/2 to that of the internal reference. **(F)** The ratio of the optical density of ERK1/2 to that of the internal reference. The data are presented as the mean ± SEM, *n* = 6; compared with the control group, **P < 0.01; compared with the DNP group, **^#^***P* < 0.05, **^##^***P* < 0.01; ns indicates a non-significant difference.

Western blot analysis was used to detect the levels of p-p38 and p38 in the DRG as an indication of the phosphorylation of proteins in the p38 mitogen-activated protein kinase (p38MAPK) signaling pathway. The electrophoretic protein bands (Figure 5A) and the ratio of the IOD of the target protein to that of β-actin (Figures 5B,C) are shown. A significantly lower level of the p-p38 protein was detected in the control group than in the DNP group (*P* < 0.01); the level of the p-p38 protein in the DNP + A438079 group was significantly lower than in the DNP group (*P* < 0.05), but no significant difference was observed between the control group and DNP + A438079 group (*P* > 0.05). Similarly, a significant difference in the level of the p-p38 protein was not observed between the DNP + NS group and DNP group (*P* > 0.05). The levels of phosphorylated proteins involved in the MAPK-ERK1/2 signaling pathway were also measured in the groups described above, and the trend in the levels of these proteins was highly consistent with that of proteins involved in the p38 signaling pathway (Figures 5D–F). This finding indicates that the P2X_7_ receptor antagonist A438079 inhibits the phosphorylation of p38 and ERK1/2, which may inhibit the expression of TRPV1 and P2X_7_ receptors in the DRGs of DNP rats.

### Molecular Docking of A438079 With the P2X_7_ Receptor and TRPV1

**FIGURE 6:**
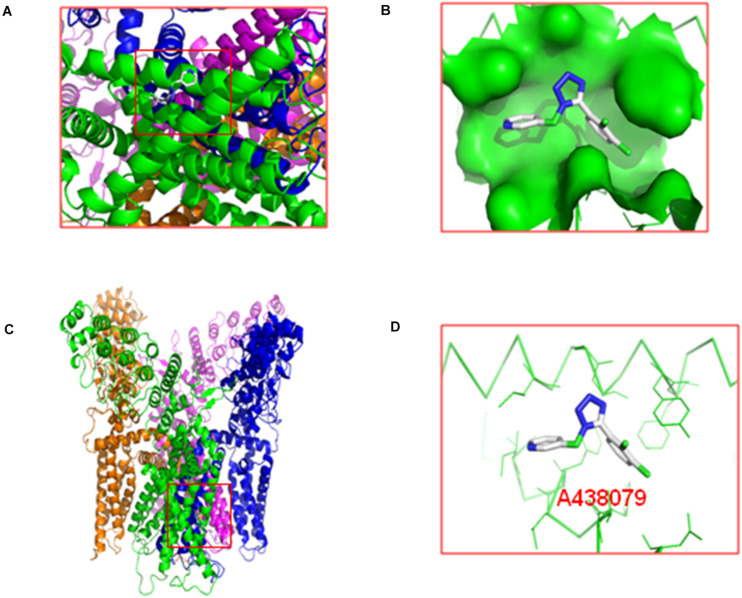
Molecular docking of A438079 with the TRPV1. **(A)**, top view. **(B)**, the docking pocket between A438079 and TRPV1. **(C)**, front view. **(D)**, A438079 does not form a hydrogen bond with TRPV1, and A438079 is in the middle.

The molecular docking of A438079 with the TRPV1 protein was performed by AutoDock4.2. The result showed that the P2X_7_ receptor antagonist A438079 does not form a hydrogen bond with TRPV1 (Figure 6). The result of this experiment indicates that the effect of A438079 on TRPV1-mediated DNP is mediated by its interaction with the P2X_7_ receptor, but this compound does not exert a direct effect on TRPV1.

## Discussion

Pain is a complex physiological process including many components, while chronic pain is a pathological state (Nazıroǧlu et al., 2020). DNP is one of the common chronic complications of diabetes and an early symptom of diabetic neuropathy, which is also related to metabolic disorders, neurotrophic disorders, and autoimmune and inflammatory responses caused by hyperglycemia (Sierra-Silvestre et al., 2018). Currently, DNP is difficult to treat, partially because the underlying mechanism of pain is not completely understood (Desai et al., 2014). For many years, pain treatment methods have focused on neurons, but with progress in research, glial cells may become a new target for pain management. Purinergic receptors can be divided into P1 and P2 receptors, and P2 receptors are further divided into ligand-gated ion channel type P2X receptors (including P2X_1–7_) and G-protein-coupled P2Y receptors (Kuan and Shyu, 2016; Burnstock and Gentile, 2018; Alarcon-Vila et al., 2019). Studies have confirmed that P2X_7_ receptors on SGCs are involved in the pathogenesis of NPP and inflammatory pain (Alarcon-Vila et al., 2019; Ni et al., 2020). Our experimental results showed that the MWT and TWL of DNP model rats were significantly reduced compared to those of control rats, indicating that the mechanical and heat hypersensitivity enhanced in DNP model rats. However, after treatment with the P2X_7_ receptor antagonist A438079, the mechanical and heat hypersensitivity decreased in DNP model rats, indicating that the P2X_7_ receptor is involved in the pathogenesis of DNP.

The transient receptor potential (TRP) channel is a non-selective cation channel that is expressed in various tissues and organs of the human body, participates in various sensory processes in the body, and plays an important role in maintaining the normal physiological function of the body (Kaneko and Szallasi, 2014; Yue et al., 2015). At least 6 TRPV subtypes, namely, TRPV1-6 (Nilius and Szallasi, 2014) have been identified to date. TRPV1-4 is heat-sensitive ion channel, while TRPV5 and TRPV6 are ion channels with a high selectivity for calcium ions (Mickle et al., 2016). Many factors cause pain, such as capsaicin and its analogs, bradykinin, ATP, TNF-α, H^+^, fatty acid derivatives, and PGE2, and can directly or indirectly upregulate the expression of TRPV1 or activate TRPV1 to cause various types of pain, particularly inflammatory pain and thermal hyperalgesia (Alawi and Keeble, 2010; Luo et al., 2011; Hsu et al., 2014; Ferreira et al., 2019), suggesting that TRPV1 is involved in the processes of inflammation and immune activation. In the present study, mRNA and protein levels of TRPV1 in DRG neurons in DNP model rats were significantly increased compared to those in control rats. The mRNA and protein levels of TRPV1 were decreased after intrathecal injection of the P2X_7_ receptor antagonist A438079, indicating that TRPV1 is involved in the hypersensitivity associated with DNP. Based on the interaction between neurons and glial cells in the sensory ganglia (Gu et al., 2010) and the results described above, we speculated that the P2X_7_ receptor may have a certain role in TRPV1-mediated DNP in rats.

GFAP is a marker of SGCs, and its upregulation indicates the activation of SGCs (Feldman-Goriachnik et al., 2018; Liu H. et al., 2018), while NeuN is a neuronal marker (Halene et al., 2016). The expression of NeuN and GFAP in the DRG was detected in our study. The results of double immunofluorescence staining showed a significant increase in the coexpression of TRPV1 and NeuN in DRG neurons from the model group compared to the control group and that the coexpression of the P2X_7_ receptor and GFAP in SGCs was also significantly increased; the coexpression of TRPV1 and NeuN and coexpression of the P2X_7_ receptor and GFAP was decreased in the model group after treatment with the P2X_7_ receptor antagonist A438079. Therefore, DRG neurons and SGCs are activated during DNP, along with the corresponding TRPV1 and P2X_7_ receptors. Additionally, the P2X_7_ receptor antagonist A438079 can inhibit the activation of P2X_7_ receptors, which can reduce the release of cytokines, etc., thereby reducing the damage to neurons and the activation of TRPV1.

Upregulation of cytokine expression is another feature of activated SGCs and may play an important role in information exchange between ganglion cells. The release of cytokines by SGCs is increased under pathological conditions, thus affecting the activity of neurons (Otoshi et al., 2010; Berta et al., 2012; Cairns et al., 2015; Afroz et al., 2019). Exciting P2X_7_ receptors can increase the release of cytokines such as IL-1β in glial cells, which cause chronic pain. Peripheral tissue inflammation significantly increased the expression of COX-1 and COX-2 in DRG cells. IL-1β induces the expression of COX-2, and subsequently increases prostaglandin synthesis in different cells including DRG cells. The prostaglandins released in DRG activate an autocrine signaling mechanism in primary afferent neurons to sensitize them and directly or indirectly activate TRPV1 to induce various pain sensations (Yaksh et al., 2001; Amaya et al., 2009; Araldi et al., 2013). Our results showed that the expression of IL-1β was upregulated and that the relative level of the IL-10 protein was decreased in the DRGs of DNP model rats compared to those of control rats, while the P2X_7_ receptor antagonist A438079 reduced the upregulation of IL-1β expression and increased the relative level of IL-10. This suggests that inflammatory factors may be involved in the pathophysiological mechanism by which P2X_7_ receptor participates in DNP mediated by TRPV1.

Additionally, the p38MAPK and ERK pathways are involved in the regulation of the pathophysiological functions of various related diseases mediated by TRPV1 (Shin et al., 2016; Hong et al., 2017) and the pathogenesis of inflammatory and neurodegenerative diseases mediated by the P2X_7_ receptor (Ji et al., 2018; Yu et al., 2018; Pérez de Lara et al., 2019). In our study, we detected changes in the levels of proteins involved in the p-p38MAPK and p-ERK1/2 signaling pathways in each group of rats using western blotting. The levels of p-p38 and p-ERK1/2 in the DNP group were increased compared to those in the control group, but the levels of these proteins in the DNP + A438079 group were significantly decreased compared to those in the DNP group. The detection of changes in the phosphorylation of cell signal transduction molecules showed that the P2X_7_ receptor antagonist A438079 inhibited the activation of p38MAPK and ERK1/2 mediated via TRPV1 and P2X_7_ receptor in DRG, which provides experimental evidence for the possible mechanism underlying the role of the P2X_7_ receptor in TRPV1-mediated DNP.

We used AutoDock4.2 for molecular docking experiments to further confirm that the P2X_7_ receptor antagonist A438079 directly binds to the P2X_7_ receptor and then interacts with TRPV1 rather than directly acting on TRPV1. The results showed that A438079 does not form a hydrogen bond with the TRPV1. Thus, A438079 modulates TRPV1-mediated DNP by binding the P2X_7_ receptor but has no direct effect on TRPV1.

## Conclusion

In summary, the P2X_7_ receptor antagonist A438079 alleviates nociception, as measured by the behavioral responses of DNP rats, and reduces the upregulation of TRPV1; activation of the P2X_7_ receptor also increases the release of IL-1β and inhibits the release of IL-10, and A438079 has no direct effect on TRPV1, suggesting that the P2X_7_ receptor is involved in the maintenance and development of DNP mediated by TRPV1, and the mechanism may involve reducing the activation of p38MAPK and ERK1/2.

## Data Availability Statement

The original contributions presented in the study are included in the article/supplementary material, further inquiries can be directed to the corresponding author.

## Ethics Statement

The animal study was reviewed and approved by the Ethics Committee of Nanchang University.

## Author Contributions

AW: methodology, validation, formal analysis, investigation, data curation, writing—original draft, writing—review and editing, and visualization. XS: methodology and writing—original draft. RYu, BQ, and RYa: methodology. CX: conceptualization, investigation, writing—review and editing, supervision, and project administration. All authors read and approved the final manuscript.

## Conflict of Interest

The authors declare that the research was conducted in the absence of any commercial or financial relationships that could be construed as a potential conflict of interest.

## Footnotes

1 https://swissmodel.expasy.org/

2 http://www.pymol.org/
